# Detection of *Mycobacterium avium* subspecies in the gut associated lymphoid tissue of slaughtered rabbits

**DOI:** 10.1186/s12917-015-0445-2

**Published:** 2015-06-11

**Authors:** Rakel Arrazuria, Iker A Sevilla, Elena Molina, Valentín Pérez, Joseba M Garrido, Ramón A Juste, Natalia Elguezabal

**Affiliations:** Animal Health Department, NEIKER-Instituto Vasco de Investigación y Desarrollo Agrario, Berreaga, 1, 48160 Derio, Bizkaia Spain; Departamento de Sanidad Animal, Facultad de Veterinaria, Universidad de León, León, Spain

**Keywords:** Animal pathogens, Epidemiology, *Mycobacterium avium* subsp, *Mycobacterium avium* complex, *Thermoactinomyces* sp, Rabbits, Slaughter

## Abstract

**Background:**

Rabbits are susceptible to infection by different species of the genus *Mycobacterium*. Particularly, development of specific lesions and isolation of *Mycobacterium avium* subsp. *avium* and *Mycobacterium avium* subsp. *paratuberculosis*, both subspecies of the *M. avium* complex, has been reported in wildlife conditions. Although, rabbit meat production worldwide is 200 million tons per year, microbiological data on this source of meat is lacking and more specifically reports of mycobacterial presence in industrially reared rabbit for human consumption have not been published. To this end, we sought mycobacteria by microbiological and histopathological methods paying special attention to *Mycobacterium avium* subsp. *paratuberculosis* in rabbits from commercial rabbitries from the North East of Spain.

**Results:**

*M. avium* subsp. *paratuberculosis* was not detected either by culture or PCR. However, *Mycobacterium avium* subsp. *avium* was detected in 15.15 % (10/66) and *Mycobacterium avium* subsp. *hominissuis* was detected in 1.51 % (1/66) of gut associated lymphoid tissue of sampled animals by PCR, whereas caecal contents were negative. 9 % (6/66) of the animals presented gross lesions suggestive of lymphoid activation, 6 % (4/66) presented granulomatous lesions and 3 % (2/66) contained acid fast bacilli. Mycobacterial isolation from samples was not achieved, although colonies of *Thermoactinomycetes* sp. were identified by 16s rRNA sequencing in 6 % (4/66) of sampled animals.

**Conclusions:**

Apparently healthy farmed rabbits that go to slaughter may carry *M. avium* subspecies in gut associated lymphoid tissue.

## Background

Rabbits have been found to be naturally susceptible to *Mycobacterium avium* subsp. *paratuberculosis* (Map) infection in the wild [1–4], to *Mycobacterium avium* subspecies infection in natural conditions, specifically *Mycobacterium avium* subsp. *avium* (Maa) in pigmy rabbits [5] and moderately susceptible to Map in laboratory conditions [6, 7].

Map and Maa are subspecies of *Mycobacterium avium*, as well as *Mycobacterium avium* subsp. *silvaticum* (Mas) and *Mycobacterium avium* subsp. *hominissuis* (Mah) [8]. From an epidemiologic point of view, Maa is typically virulent for birds and small terrestrial mammals causing a range of lesions that go from characteristic tuberculous lesions in parenchymatous organs, to lymphadenitis and disseminated infection [9–11]. Mah has the human [12] and the pig [13] as its primary hosts and it is frequently found in soil [14]. Map is the causal agent of paratuberculosis in ruminants and it has been controversially associated with human inflammatory bowel disease, more precisely with Crohn’s disease [15–17] and also diabetes mellitus [18]. Its prevalence in slaughtered cattle in Europe has been estimated to be up to 50 % [19]. Mas has been isolated from wood pigeons [20], roe deer [21] and horses causing tuberculous-like lesions in these animals [22]. However, since there is a controversy about the real existence of Mas as a unique subspecies independent of Maa [23], for the present work we will consider Mas as part of Maa, and jointly refer to them as Maa/Mas.

*Mycobacterium avium* subspecies members are widely spread in the environment and often enter in contact with animals and humans. Transmission from animals to humans can occur either through the consumption of contaminated foods or *via* direct contact with an infected animal. Apparently healthy animals thereby may represent a reservoir for *Mycobacterium avium* subspecies and these pathogens may enter the food chain during slaughter. Most work focused on the detection of *M. avium* subspecies in animal species for human consumption has been performed on meat products. Map has been detected in beef, pork and chicken [24], whereas Mah has been detected in beef, pork and lamb [24]. Detection of non-tuberculous mycobacteria or *Mycobacterium avium* subspecies at slaughter has been described in lymph nodes of pigs [25–27] and detection of Map has been reported in lymph nodes, muscle and faeces of both dairy and beef cattle [28].

*Mycobacterium avium* subspecies presence in rabbits in wild conditions led us to hypothesize that these mycobacteria could also be present in commercial rabbits that go to slaughter and thus represent a route of exposure for humans. The aim of the present study was to carry out a small survey on the frequency of mycobacterial microorganisms detected by solid and liquid culture and by a tetraplex real-time PCR for *Mycobacterium* genus, *M. avium* subspecies and *M. tuberculosis* complex in gut associated lymphoid tissue (GALT) and caecal contents of apparently healthy rabbits at slaughter.

## Methods

### Ethics statement

Animals used in this study did not undergo any manipulation prior to stunning for standard industrial slaughter according to the pertinent legislation. For this reason, no specific ethical approval was required.

### Animals and sampling

We contacted the official veterinarian of the nearest rabbit slaughterhouse to set-up a sampling schedule. This study was based on samplings that took place from May to October of 2013 (decontamination procedure set-up and PCR evaluation) and during January and February of 2014 (proper study) in a rabbit slaughterhouse (Basque Country, Spain) processing 1300000–1400000 rabbits annually. Rabbits are typically slaughtered with an average age of two months and average live weight around 1.8-2.2 kg (http://www.magrama.gob.es/es/ganaderia/temas/produccion-y-mercadosganaderos/INDICADORES_ECONÓMICOS_SECTOR_CUNÍCOLA_2013_tcm7-330314.pdf). Breeding rabbits are slaughtered at about 2–2.5 years of age. Production rabbits are often outbred in order to maximize meat production [29]. This slaughterhouse was operated by an agriculture cooperative company and complied with the pertinent Basque (Basque Government Decree 454/1994), Spanish (Spanish Government Law 32/2007 and Royal decree 731/2007) and European (Council Regulation (EC) No 1099/2009) legislation on animal welfare under the supervision of official veterinarians and the samples obtained were authorized by the slaughterhouse managers.

A total of 12 animals (decontamination procedure set-up and PCR evaluation) and 66 animals (proper rabbit slaughterhouse study) from 21 farms scattered in 6 provinces of North-East Spain were sampled. Samples were drawn from two young animals from each batch of sacrifice and from a third additional breeding animal, if the batch included breeding rabbits. Samples were kept in refrigeration for 6-12h before being processed.

### Decontamination procedure

In order to determine the optimum NaOH concentration for decontamination initial set-up experiments were performed. Mucosa from sacculus rotundus and vermiform appendix from 12 rabbits were used to test decontamination with 2 %, 4 %, 6 % and 8 % NaOH. Decontamination procedures were run for 10 and 15 min. Contamination was observed in both solid and MGIT culture at NaOH concentrations lower than 6 % for both 10 and 15 min. 6 % NaOH for 15 min was the lowest concentration that resulted in no contaminated samples. For this reason, the final decontamination procedure was performed with these conditions. Also manufacturer’s instructions do not recommend running decontamination for more than 15 min or NaOH concentrations higher than 6 % (BACTEC™ MGIT ™960).

10 ml of sterile water were added to 2 g of sample tissue. Homogenization was then performed on a Stomacher blender for 1 min at medium speed. Afterwards, the homogenized solution was transferred to a tube and 10 ml of 6% NaOH were added. After a vortex mix, the suspension was incubated for 15 min at room temperature before neutralization with 15 ml of phosphate buffer. The suspension was mixed well and centrifuged for 20 min at 2,885 × g and the supernatant was discarded. Pellets were suspended in 2 ml of sterile water.

### Culture

#### Solid culture

Four drops (150 μl)/per tube of the decontaminated suspension were seeded on solid media: Herrold’s Egg Yolk Medium (HEYM); Middlebrook 7H10 with penicillin, amphotericin B, and chloramphenicol; Lowenstein-Jensen with penicillin, amphotericin B and supplemented with mycobactin J and Tsukamura minimal media with cycloheximide [30]. All seeded tubes were incubated at 37+/−1 °C and checked for growth at 8, 12, 16 and 20 weeks. After seeding on solid culture, the remaining inoculum was used for liquid culture.

#### Liquid culture

Pellets destined to liquid culture were seeded on BBL Mycobacteria Growth Indicator Tubes (MGIT) supplemented with BACTEC MGIT growth supplement and BBL MGIT PANTA (Becton, Dickinson and Company). Tubes were incubated for 45 days in a BACTEC MGIT 960 System and time to detection (TTD) values were recorded.

### PCR evaluation

PCR performance on spiked mucosa was evaluated since this specimen is known to be tricky because of the presence of PCR inhibitors.

#### Inocula preparation for tissue spiking

Mah Strain 104 was grown in Middlebrook 7H9 (M7H9) broth supplemented (v/v) with 10 % Middlebrook OADC enrichment (Becton, Dickinson and Company, MD, USA), 0.2 % glycerol and 0.05 % Tween 80 (Sigma-Aldrich, Co. Ltd., Haverhill, UK). Mycobacteria were harvested by centrifugation at 2,800 × g. Pellets were washed twice in phosphate buffered saline (PBS). Mycobacteria were resuspended in PBS containing 0.2 % glycerol and 0.05 % Tween 80 (PBS-GT). Bacterial suspensions were adjusted to 1 McFarland unit after turbidity measurements were performed with a Densicheck densitometer (Bio-Mérieux, Marcy L’Etoile, France). Five ten-fold dilutions of the bacterial suspensions were prepared in PBS-GT and the highest, medium and lowest, with a 2 log difference between each of them were used to inoculate negative tissues. To verify the viable number of CFU, the inocula were plated onto appropriately supplemented agar-solidified M7H9 flasks.

#### Mucosa spiking

Mucosa was scraped from vermiform appendix tissue from 12 apparently healthy rabbits previously confirmed to be negative for the presence of mycobacteria by PCR, culture and histopathology and it was thoroughly mixed and homogenized. The blended tissues were divided in batches of 1 g of mixture per stomacher bag. The stomacher bags were inoculated with 100 μl of one of each of the previously adjusted bacterial suspensions (high, medium and low). 300 mg of the homogenate spiked tissues were extracted as described in the DNA extraction section and MycMavMtc PCR [31]. In cases where PCR inhibition took place ½ and ¼ dilutions of the extracted DNA were performed previous to PCR reaction and this showed to be enough to prevent inhibition.

### S**laughterhouse sample study**

**Fig. 1:**
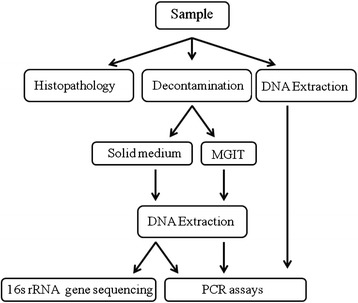
Experimental scheme showing methods followed at each stage

A total of 66 animals (48 young and 18 breeding animals) were sampled and different steps that are summarized on Fig. 1 were taken to investigate the specimens.

Briefly, sacculus rotundus and vermiform appendix were scraped for mucosa and caecal contents were collected. Part of each sample was used for direct molecular detection and part for decontamination and subsequent culture. Based on set-up results, the final decontamination procedure was performed with 6 % NaOH as described in the decontamination procedure section because it was the lowest concentration found to inhibit contamination. Both solid and liquid cultures were performed followed by PCR confirmation and sequencing of the 16Sr RNA gene when non-mycobacterial colonies were isolated.

Histopathological analysis was performed on samples of sacculus rotundus and vermiform appendix of a total of 22 rabbits (14 young rabbits and 8 breeding rabbits) including animals with and without macroscopic lesions.

### DNA extraction

#### Spiked tissues and fresh rabbit slaughterhouse samples

DNA extraction was performed on spiked mucosa (PCR evaluation), sacculus rotundus, vermiform appendix and caecal content (slaughterhouse study), using DNA Extract-VK (Vacunek S.L, Bizkaia, Spain) following manufacturer’s instructions with modifications. Briefly, 300 mg of tissue or caecal content were weighed in a microtube. 250 μl of sterile distilled water, plus 250 μl of VK-Lysis buffer and 300 mg of VK-extraction beads were added to the tissue and homogenized at 30Hz during 10 min on TissueLyser II (Qiagen). Following homogenization, samples were centrifuged at 7,000 × g for 5 min and 200 μl of the supernatant transferred to a new vial. 25 μl of Proteinase K were added to the previous solution and tubes were incubated at 56 °C for 15 min. Lysis was performed adding 200 μl of Lysis Buffer VL-LB3 to the previous solution and mixing thoroughly. The solution was incubated at 70 °C for 10 min. Then, 210 μl of ethanol (96-100 %) were added and mixed thoroughly. The final mixtures were loaded on VK-DNA binding columns and centrifuged at 11,000 × g for 1 min. Pass-through liquid was discarded. Washing steps were performed with 500 μl of Wash Buffer VK-WB1 and 600 μl of Wash Buffer VK-WB2 with a 11,000 × g 1 min centrifuging step in between. After washes were done, 100 μl of Elution Buffer were added to the column and the column left for 1 min. DNA was recovered by centrifuging at 11,000 × g for 1 min. Extracted DNA was stored at −20 °C until PCRs were performed. Negative DNA extraction controls (for every 23 samples), no template and positive PCR controls (for every PCR) were included in all downstream PCR assays.

#### Solid medium isolated colonies

A loopful of cells from each colony growing on solid medium was placed in 100 μl of sterile water. Vials were incubated at 95 °C for 10 min and centrifuged at 13,800 × g for 5 min. The supernatant was used as template for further PCR assays.

#### Positive MGIT liquid medium

One millilitre of vortexed liquid culture from the MGIT tubes was collected and centrifuged at 13,800 × g for 5 min, the supernatant was discarded and after a washing with 1 ml of sterile distilled water, the pellet was suspended in 250 μl of sterile water. Then 300 mg of zirconium beads were added and homogenized at 30Hz for 10 min on TissueLyser II (Qiagen). Following homogenization, samples were centrifuged at 7500 × g for 15 min and the supernatant was collected.

### Molecular detection

#### Detection of mycobacteria by Tetraplex real-time PCR (MycMavMtc)

For initial screening purposes to test the presence of *Mycobacterium* sp., a fourplex real-time PCR described by Sevilla *et al.* [31] was performed. This PCR is able to detect the genus *Mycobacterium* and then distinguish between the *M. tuberculosis* complex and *M. avium* subspecies. The target genes were the internal transcribed spacer (ITS) between 16S and 23SrRNA genes, insertion sequence IS1311 and the devR genr for *Mycobacterium* genus, *M. avium* subspecies and *M. tuberculosis* complex members respectively.

Reactions were carried out in a final volume of 25 μl containing 5 μl of template DNA, and 1X TaqMan Universal PCR MasterMix without AmpErase uracil-DNA glycosylase (Applied Biosystems), 0.3 μM of each primer, 0.2 μM of each probe. An internal amplification control (IAC) was included in the reaction to rule out false negative results due to inhibitors. ROX (6-carboxyl-X-rhodamine) dye was used as passive reference reporter. Amplification was carried out in a 7500 Real-Time PCR instrument (Applied Biosystems) and consisted of one denaturation and polymerase activation cycle of 10 min at 95°C, and 45 cycles of denaturation at 95 °C for 15 s and annealing/extension at 60°C for 1 min. Results were analyzed using the 7500 System SDS software v. 1.4 (Applied Biosystems). Threshold cycle (*C*_*T*_) and baseline were automatically determined by the software and verified by visual examination of the threshold line in amplification plots.

#### *M. avium* subsp*. paratuberculosis* detection by IS*900* & IS*Map02* real-time PCR

To identify Map positive samples, a real time multiplex PCR described by Sevilla *et al.* [32] was performed. This PCR detects IS*900* and IS*Map02* DNA sequences of *M. avium* subsp. *paratuberculosis*.

Briefly, the reaction mixture contained 1× TaqMan Universal PCR Master Mix (Applied Biosystems), 300–400 nM of each primer, 200 nM of each probe, and 5μl of DNA extract in a final volume of 25 μl. An IAC is also included in the PCR reaction.

Amplification and real time measurement were performed in the same system and conditions as described in the previous PCR protocol.

#### *M. avium* subsp. *hominisuis* and *M. avium* subsp. *avium/M. avium* subsp. *silvaticum* detection by IS*1245* & IS*901* PCR

In order to identify other *M. avium* subspecies, a previously published real time PCR was carried out, based on the detection of IS*901* and IS*1245* insertion sequences [33]. Maa/Mas organisms are positive for IS*1245* and IS*901*, while Mah is only positive for IS*1245*. The PCR did not include DNA concentration standards for quantification purposes because the objective was just to detect and identify Mah and Maa/Mas.

The final reaction mixture contained the same PCR ingredients at the same concentration indicated above for Map PCR but primers and probes were those previously described by Slana *et al.* [33]. Amplification and real time measurement were performed in the same system and conditions as described in the previous PCR setting.

### 16S rRNA gene sequencing of colonies on solid medium

The identification of colonies grown on solid media that were not mycobacteria as assessed by previously described PCR methods was performed by 16S rRNA gene sequencing. Amplification of target DNA was performed by PCR using bacterial universal primers pAF (5′- AGA GTT TGA TCC TGG CTC AG-3′) and 530R (5′-CCG CGG CKG CTG GCAC-3′). Amplification was carried out in a 2700 GeneAmp PCR Instrument (Applied Biosystems) and consisted of one denaturation and polymerase activation cycle of 4 min at 94 °C, and 30 cycles of denaturation at 94 °C for 30 s, annealing at 50 °C for 30 s and extension at 72 °C for 40 s. The PCR product was cleaned with Illustra Exoprostar 1-Step (GE Healthcare Life Sciences) following manufacturer’s instructions.

The sequencing reaction was carried out with BigDye® Terminator v3.1 cycle sequencing kit (Applied Biosystems) and resolved with a 3130 ABI *(*Applied Biosystems*)* capillary sequencer. Sequencing conditions consisted of 30 cycles of denaturation at 96 °C for 10 s, annealing at 50 °C for 5 s and extension at 60 °C for 4 min. Sequence alignment was performed using BLAST (Basic Local Alignment Search Tool) and identification with 100 % of coverage and 99-100 % of identity was made.

### Gross pathology

The whole digestive system of the rabbits was inspected for gross anatomical change. Samples from sacculus rotundus and vermiform appendix were collected for solid and/or liquid culture and histopathological analysis. Samples from sacculus rotundus, vermiform appendix and caecal contents were collected for detection of mycobacteria by PCR.

### Histopathology

Histopathological analysis was performed on samples of sacculus rotundus and vermiform appendix and on other samples that presented macroscopic lesions. The tissues were fixed in 10 % neutral buffered formalin for a minimum of 24 h, trimmed, dehydrated through graded alcohols, embedded in paraffin wax and sectioned at 5μm. These sections were mounted on glass slides stained with haematoxylin and eosin (HE) or by the Ziehl-Neelsen (ZN) method for acid fast bacteria (AFB) according to standard procedures. Slides were examined under the microscope for granuloma formation and AFB presence.

### Statistical analysis

Association of mycobacteria detection and age was tested with the Chi square or Fisher exact probability (FET) tests for the frequencies of PCR results according to age using the PROC FREQ of the SAS statistical package (SAS Institute Inc., Cary, NC, USA). For all analyses, a p value of <0.05 was considered to be statistically significant.

## Results

### PCR evaluation

MycMavMtc PCR results of the spiked rabbit mucosa are shown in Table 1. Inhibition was observed at all bacterial concentrations when samples were not diluted and only probe IS*1311* was detected in the highest concentration. After dilution steps were performed, inhibitory effects were mitigated. In the medium concentration bacterial suspension a ½ dilution was enough to detect *M. avium* sp. DNA by the IS*1311* probe. However, in the low concentration inoculum after ½ and ¼ dilutions the IS*1311* probe was not positive and only the internal amplification control gave significant C_*T*_ values.

**Table 1:** M. avium subsp hominissuis spiked rabbit mucosa C_T_s of MycMavMtc PCR

		Mah spiked rabbit mucosa		
		high	medium	low
Dilution	DNA Probe	7.66×10^6^ (CFU/g)	7.66×10^4^ (CFU/g)	7.66×10^2^ (CFU/g)
1/1	ITSdenak	UD	UD	UD
	IS*1311*	31.77	UD	UD
	IAC	UD	UD	UD
1/2	ITSdenak	43.56	UD	UD
	IS*1311*	28.5	36.66	UD
	IAC	35.1	41.19	36.00
1/4	ITSdenak	39.7	UD	UD
	IS*1311*	28.85	34.50	UD
	IAC	32.62	33.99	33.63

Mah: *M. avium* subps. *hominissuis*, ITSdenak: detects *Mycobacterium* genus, IS*1311*: detects *Mycobacterium avium*, IAC: internal amplification control, UD: undetermined no threshold-crossing fluorescence, devR detecting Mtb complex members was UD for all assayed conditions

### Slaughterhouse sample study

#### Gross pathology and digestive system parameters

**Fig. 2:**
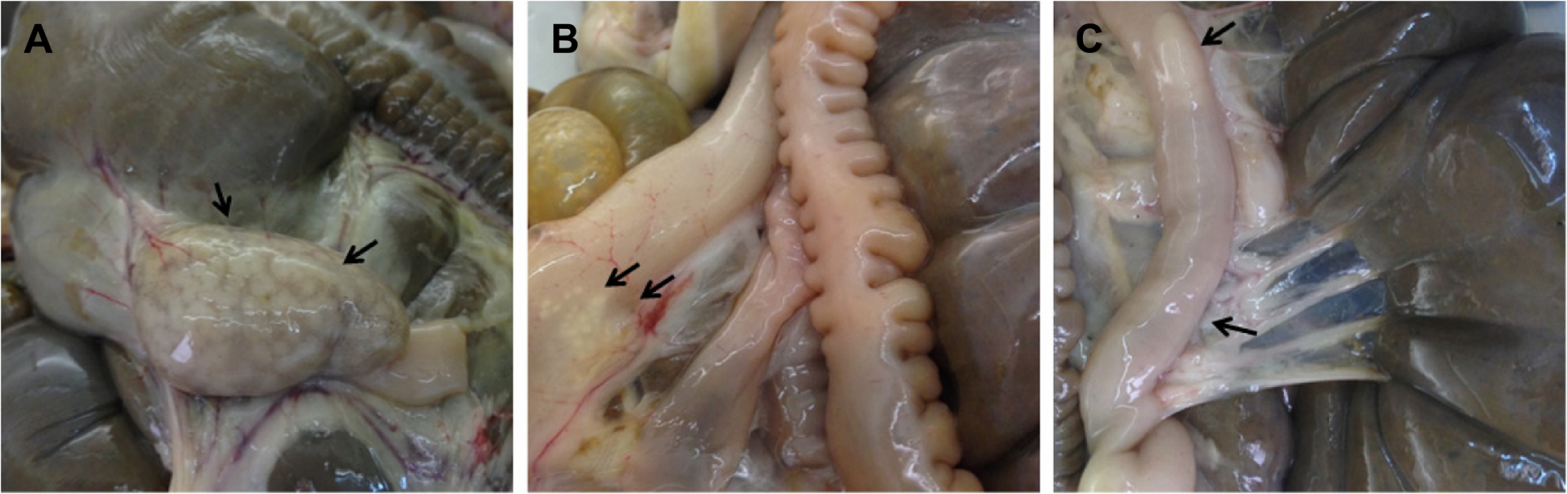
Macroscopic lesions found in slaughtered rabbits. **a** sacculus rotundus with pale white spots and thickened wall (animal D2.B2), **b** vermiform appendix with white spots and thickened wall (animal E1.Y2), **c** ileum with thickened walls and a reactive Peyer’s patch (animal C1.Y2)

Of the 66 necropsied animals, 6 (9 %) showed visible pathological lesions consistent with mycobacterial infection. They were characterized by the presence of focal thickenings of the intestinal wall in the sacculus rotundus and vermiform appendix together with the presence of pale-whitish spots consistent with lymphoid follicle hyperplasia. Jejunal and ileal wall thickening was observed in one and three animals respectively, affecting some areas of the intestine in these regions. Representative photographs of lesions observed in three different animals are shown in Fig. 2.

### Molecular detection

Of the 66 analyzed rabbits, 11 were positive for *M. avium* in GALT (16.66 %). Of these, six animals (54.54 %) were positive in vermiform appendix only, 2 (18.18 %) in sacculus rotundus only, and 3 (27.27 %) in both tissue types. C_*T*_s for IS*1311* probe were 36.48+/−2.32 for undiluted samples and 37.12+/−1.01 for diluted samples. Attending to tissue type, C_*T*_s were 36.94+/−0.83 for vermiform appendix and 35.83+/−3.88 for sacculus rotundus. Mycobacteria were not detected by PCR in caecal contents.

The 14 *M. avium* positive samples were further analyzed by IS*900* & Is*Map02* PCR for Map detection and IS*1245* & IS*901* PCR for differentiation between Maa/Mas and Mah*.* All these samples were negative for the first real-time PCR, meaning that Map was not detected in these rabbits. In contrast 13 of the *M. avium* positive samples were identified as Maa/Mas and 1 was identified as Mah according to the results obtained with IS*1245* & IS*901* PCR (Table 2).

**Table 2:** Gross pathology and molecular detection of sacculus rotundus and vermiform appendix from analyzed slaughtered rabbits

		Gross pathology	Molecular detection	
	Tissues (n)	Animals (n (%))	Animals (n (%))	Identity
**Young rabbits**	SR (48)	2^a^ (11.1)	3 (4.2)	Maa/Mas
			1 (2.0)	Mah
	VA (48)	1 (5.5)	7 (14.6)	Maa/Mas
**Breeding rabbits**	SR (18)	3^b^ (16.6)	2 (11.1)	Maa/Mas
	VA (18)	0 (0)	1 (5.5)	Maa/Mas

n: number of tissues or animals, %: percentage of positive results, SR: sacculus rotundus, VA: vermiform appendix, Maa: *M. avium* subsp*. avium*, Mas: *M. avium* subsp *silvaticum*, Mah: *M. avium* subsp *hominissuis* ^a^Lesions spread to the ileum in both animals, ^b^Lesions spread to the ileum in one animal

### Culture

The culture of samples in BACTEC MGIT recorded contamination of 19.69 % of sacculus rotundus and 7.5 % of vermiform appendix samples. The remaining samples gave TTD readouts compatible with mycobacterial growth patterns on an average of 7.36 days for samples of sacculus rotundus and 6.95 days for samples of vermiform appendix but with a negative result in the MycMavMtc PCR. As a consequence, all MGIT cultures were finally recorded as contaminated.

No contamination on solid media was observed. Four samples cultured on Lowenstein-Jensen solid medium yielded colonies that were negative in the MycMavMTC. Sequencing of 16S rRNA gene identified them as *Thermoactinomycetaceae bacterium W8742* in three cases and *Thermoactinomyces sanguinis* in one case (Table 3).

**Table 3:** Molecular analysis, gross pathology and histopathology results of animals with at least one positive result in any of the methods

MycMavMtc PCR			Identity	16s rRNA gene sequencing	Gross pathology	Histopathology
Animal	SR	VA				
E1.Y1	-	-	-	*Thermoactinomyceteae* ^a^	-	SR (0)/ VA (0)
E1.Y2	-	+	Maa/Mas	NI	VA	SR (0)/ VA (0)
B1.B1	-	-	-	*Thermoactinomyceteae* ^b^	-	NA
B1.B2	-	-	-	NI	SR	SR (0)/ VA (0)
E2.Y2	-	-	-	NI	SR/IL^c^/JE^c^	SR (3)/ VA (0)/ IL (1)/ JE (0)
C1.Y2	+	+	Maa/Mas	NI	SR/IL^c^	SR (0)^d^/ VA (1)/ IL (1)
C2.Y1	-	+	Maa/Mas	NI	-	SR (1)/ VA (3)
C2.Y2	-	+	Maa/Mas	NI	-	SR (0)/ VA (0)^d^
B2.Y2	-	-	-	*Thermoactinomyceteae* ^a^	-	SR (0)/ VA (0)
A1.Y2	-	+	Maa/Mas	NI	-	NA
E3.Y1	-	+	Mah	NI	-	NA
E3.Y2	-	+	Maa/Mas	NI	-	NA
B7.Y1	-	-	-	*Thermoactinomyces sanguinis* ^b^	-	NA
B5.B1	+	+	Maa/Mas	NI	-	NA
D2.Y2	+	+	Maa/Mas	NI	-	NA
D2.B1	-	-	-	NI	SR/IL^c^	SR (2)/ VA (0)/ IL (1)
D2.B2	+	-	Maa/Mas	NI	SR	SR (2)/ VA (0)
D3.Y1	+	-	Maa/Mas	NI	-	NA

SR: sacculus rotundus, VA: vermiform appendix, IL: ileum, JE: jejunum, NI: no isolation. ^a^Identified from a colony isolated from sacculus rotundus, ^b^Identified from a colony isolated from vermiform appendix. ^c^intestinal wall thickening, NA: Not analyzed. (0): Without lesions, (1): Reactive hyperplasia, (2) Unspecific granulomatous lesions (3): Granulomatous lesions,^d^: Ziehl Neelsen positive

### Histopathology

Most significant lesions were found in the sacculus rotundus and vermiform appendix lymphoid tissue. In some cases, they were formed by well demarcated granulomas compatible with mycobacterial infection, composed of between 10–50 macrophages with occasional multinucleated Langhan’s type giant cells, located in the interfollicular areas of the lymphoid tissue. In other animals, granulomatous lesions were categorized as unspecific and were formed by small granulomas, also seen in the interfollicular areas of lymphoid tissue, with less than 15 macrophages, harbouring variable amounts of a brown pigment in their cytoplasm. Microscopical examination of tissues revealed that wall thickening observed on ileum and jejunum corresponded to reactive lymphoid follicles in the ileal and jejunal Peyer’s patches. Remaining samples were classified as reactive hyperplasic or absence of lesion when tissues presented a normal form. AFB were detected on two samples that were classified as absence of lesion and were also PCR positive. Complete results for all animals are shown in Table 3.

### Association analysis

Analysis of age and gross pathology results showed that 6 out of 48 (6.25 %) of the young rabbits and 3 out of 18 (16.7 %) breeding rabbits showed some gross changes. However, these frequencies were not statistically significant (FET *p* = 0.333).

Microbiological results showed that 9 of 48 (18.7 %) young rabbits and 2 of 18 (11.1%) breeding animals were *M. avium* subspecies positive in the PCR tests, but again, these frequencies were not statistically significant (FET *p* = 0.713).

Worth mentioning is that 50 % (2/4) of the samples that presented focal lesions were PCR positive.

**Fig. 3:**
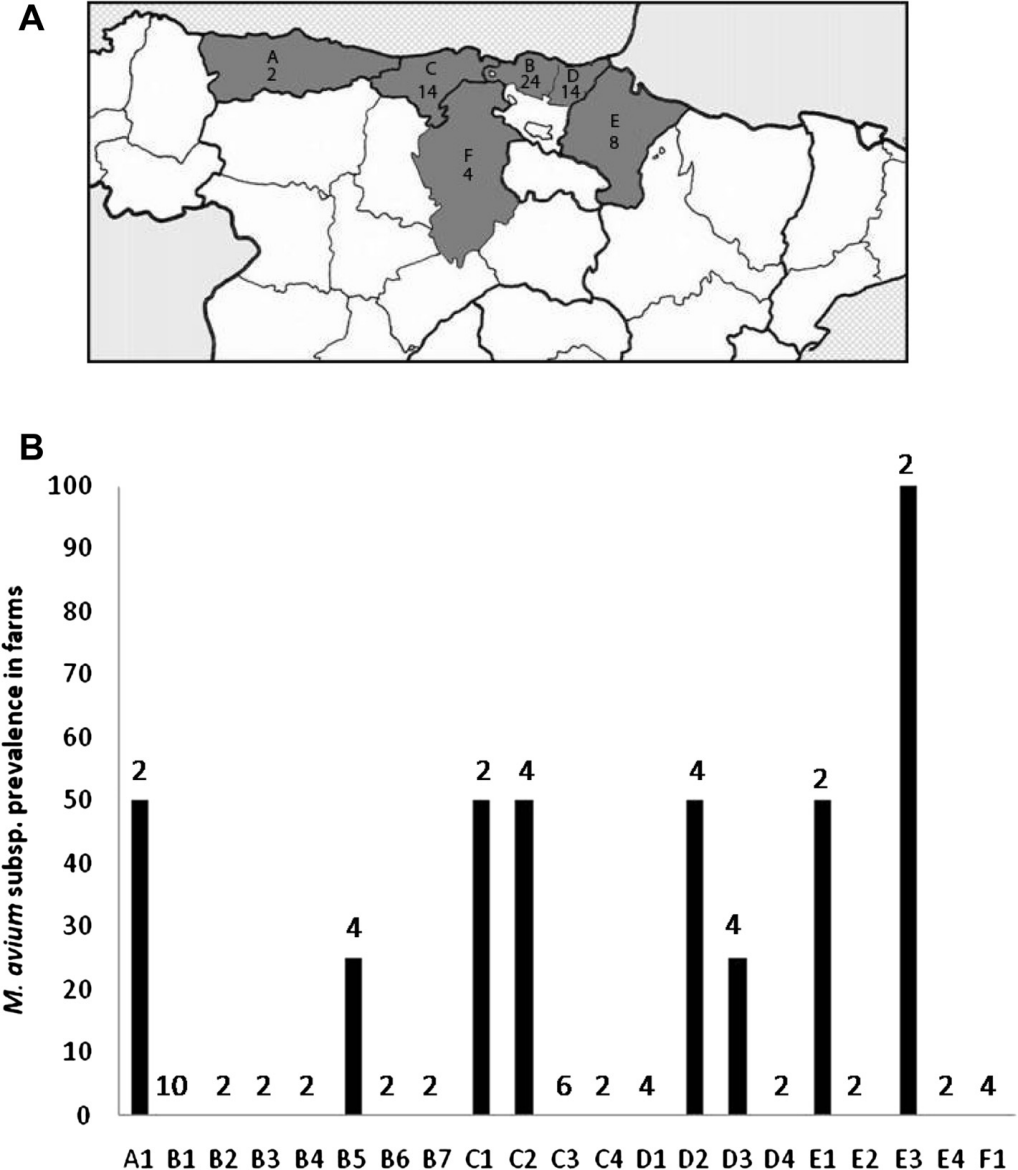
**a** Geographical origin of sampled slaughtered rabbits. A: Asturias, B: Bizkaia, C: Cantabria, D: Gipuzkoa, E: Navarra, F: Burgos and number of analyzed animals per area. **b** *M. avium* subspecies positivity of each farm identified as letters (A-F) representing each geographical area and a number (1–7) identifying the farm. Black bars represent prevalence (%), numbers on bars represent total number of analyzed animals from each farm

The geographical origin of sampled rabbits is shown on Fig. 3a. All geographical areas except for F presented at least one positive farm as shown on Fig. 3b. 8 out of 21 farms were positive (38 %), when positive was considered as having at least one *M. avium* subspecies PCR positive animal*.* Noticeable, is geographical area E where only 2 animals were sampled and one was positive for Maa and the other for Mah.

## Discussion

There is not much information in the veterinary literature concerning microbiological findings from rabbits at slaughter. A few studies have assessed *Listeria*, *Salmonella*, and *E.coli* among others [34, 35]. It is not surprising then, that data from mycobacteria and more specifically *M. avium* subspecies is scarce and mainly limited to other animal species such as cattle [28] or pigs [26, 27]. Therefore, to the best of our knowledge, this is the first study reporting *M. avium* subspecies detection in slaughtered rabbits.

In rabbits, apart from mesenteric lymph nodes and Peyer’s patches, the GALT which accounts for 50 % of the lymphoid tissue includes two specific structures: the sacculus rotundus, and the vermiform appendix. Both tissue types present M cells [36] which actively uptake and present particular antigens and microorganisms to the immune cells of the lymphoid follicle to induce an effective immune response [37]. Map has been reported to enter the domes of the Peyer’s patches through M cells [38] so orally ingested mycobacteria should end up in these sites and/or in caecal content and therefore we chose these specimens for our analysis.

Regarding PCR evaluation, the possibility of inhibition was the main concern. MycMavMtc PCR performed well at 10^4^-10^6^ CFU/g of tissue. According to our results if we had +/−10^2^ CFU/g of mycobacteria we would probably not detect them at least in our tissue type because we would necessarily have to perform dilution steps. The detection limit for this PCR for *M. avium hominissuis* in a mixture of bovine tissues has been reported as 1.59×10^2^ CFU/g [32] which is in near agreement with our findings. We must add that the DNA extraction methods of both studies are not coincident and that the bovine tissue mixture may present a more favorable chemistry for the DNA extraction and PCR reaction compared to rabbit mucosa. In any case, for the slaughterhouse study we decided to dilute template DNA if inhibition took place, maybe missing positive samples in return.

In the slaughterhouse study, although gross pathology compatible with mycobacterial infection was seen, histological analysis of GALT did not reveal presence of granulomas in all gross pathology positive animals and AFB detected by ZN were only found in two cases. The presence of granulomas in GALT associated to Mah have been reported before in sheep [39]. The unspecific granulomas may not be due to infectious agents and they may be “garbage” granulomas or on the other hand they can be ancient granulomas that accumulate waxy pigments that have been attributed to mycobacteria in previous studies [40].

DNA of *M. avium* was present in 16.6 % of the analyzed GALT samples. If we compare C_*T*_ values in sampled tissues with the PCR of artificially spiked tissues we can estimate that the bacterial load is probably below 10^4^ CFU/g. In an experimental infection in rabbits with Map, isolation was variable, since some sacculus rotundus and vermiform appendix samples that had bacterial loads of 10^2^-10^6^ genomic equivalents/g estimated by qPCR gave positive solid culture results, whereas the same tissue site samples from other animals with 10^1^-10^5^ genomic equivalents/g gave negative culture results (unpublished observations). Of course, isolation variability can be affected by the irregular distribution of AFB in the analyzed tissues among other factors. As for, liquid cultures, they were eventually contaminated and other bacteria could have used up the nutrients necessary for mycobacterial growth. MycMavMtc PCR of MGIT cultures was negative meaning that mycobacterial load was possibly under 1,59×10^2^ CFU/g, since this was the reported detection limit for MGIT culture in a bovine tissue mixture using 2 % NaOH [31]. If more than 2 % NaOH were to be used as in the present study (6 % NaOH), it would probably affect mycobacterial viability and interfere with isolation in both solid and liquid media. In solid media, only *Thermoactinomyces*, bacteria were isolated. *Thermoactinomyces* species have been implicated as causal agents of farmer’s lung diseases [41] since the disease appears in farmers that are in direct contact with mouldy hay and cereal grains where these bacterial species are known to be abundant. Also they are found in soil, rivers and dairy products [42]. We are not sure about the meaning of this finding but it could be that these bacteria are present in the grains that the rabbits are fed or in the straw litter that maybe used for nesting before kindling. In any case, it is worth mentioning that these bacteria survive decontamination techniques used for mycobacterial culture and that no macro or microscopic signs of pathology in the digestive system were detected in the animals these bacteria were isolated from.

Association analysis revealed that neither gross pathology nor mycobacterial presence was affected by age. Maa has been detected before in rabbits that had been housed with infected pigeons [43] or with doves, ducks and chickens [44] suggesting in both cases that transmission had probably occurred from infected fowl to rabbits. In our case, although not specifically examined, given the standard production housing characteristics in the studied region, it is most probable that rabbits did not have direct contact with fowl. In any case, young rabbits do present Maa implicating an early contact in their life that might point to water or feeding.

The results from this study show that *M. avium* subspecies are widely spread and in contact with farmed rabbits since only 2–3 animals analyzed from each batch of 21 farms has shown 16.6 % positivity among animals and 38 % positivity among farms. Detection of mycobacterial DNA was achieved in 50 % of the tissues with focal lesions, slightly higher than what has been reported for Map in cattle [40, 45]. At this point, we are not sure about the real significance of detecting *M. avium* subspecies in GALT tissue of slaughtered rabbits either from an animal pathological and epidemiological or Public Health perspective. Although detected granulomas suggest the generation of lesions, the focal nature of these granulomas could be compatible with a latent and controlled infection. Rabbit GALT does not enter the food chain, but if it occurred, properly cooked affected parts should not pose a risk to healthy or immunocompromised individuals. Scarcity of previous reports indicates that the problem probably does not compromise rabbit production to a big extent. Lack of other information on the infected animals such as clinical status or weight at slaughter impedes drawing conclusions. It is noteworthy to state that no Map was detected or isolated, therefore indicating that its presence in wild rabbits might be related to environmental factors that do not occur in more controlled farm conditions or that its prevalence is lower and a higher number of animals should be tested.

## Conclusions

This study should be considered as a preliminary survey that might draw attention to a hitherto undetected potential problem. Future studies should consider including other intestinal sites, associated lymph nodes and respiratory tissues as well.

The detection MAC bacteria other than Map indicates that a niche for this group of mycobacteria does exist in farmed rabbits.

## Abbreviations

Map: *Mycobacterium avium* subsp. *paratuberculosis*
Maa: *Mycobacterium avium* subsp. *avium*
Mah: *Mycobacterium avium* subsp. *hominissuis*
MAC: *Mycobacterium avium* complex
AFB: Acid fast bacilli
GALT: Gut associated lymphoid tissue
HE: Haematoxylin and eosin
ZN: Ziehl-Neelsen
HEYM: Herrold’s Egg Yolk Medium
PBS: Phosphate buffered saline
PBS-GT: PBS 0.2 % glycerol 0.05 % Tween 80
MGIT: Mycobacteria Growth Indicator Tubes
TTD: Time to detection
FET: Fisher exact probability
CFU: Colony forming unit
PCR: Polymerase chain reaction
*C*_*T*_: Threshold cycle
IAC: Internal amplification control
ROX: 6-carboxyl-X-rhodamine
UD: Undetermined
NI: No isolation
NA: Not analyzed
SR: Sacculus rotundus
VA: Vermiform appendix
IL: Ileum
JE: Jejunum
